# The Use of Harmonic Scalpel for Free Flap Dissection in Head and Neck Reconstructive Surgery

**DOI:** 10.1155/2012/302921

**Published:** 2012-05-20

**Authors:** Sebastien Albert, Charles Guedon, Caroline Halimi, Jean Pierre Cristofari, Beatrix Barry

**Affiliations:** ^1^Department of Head and Neck Surgery, Bichat University Hospital, AP-HP, 46 rue Henri Huchard, 75018 Paris, France; ^2^Service de Chirurgie Cervico-Faciale, GHU Paris Nord, Val de Seine, Hôpital Bichat-Claude Bernard, AP-HP, 46 rue Henri Huchard, 75018 Paris, France

## Abstract

Surgeons conventionally use electrocautery dissection and surgical clip appliers to harvest free flaps. The ultrasonic Harmonic Scalpel is a new surgical instrument that provides high-quality dissection and hemostasis and minimizes tissue injury. The aim of this study was to evaluate the effectiveness and advantages of the ultrasonic Harmonic Scalpel compared to conventional surgical instruments in free flap surgery. This prospective study included 20 patients who underwent head and neck reconstructive surgery between March 2009 and May 2010. A forearm free flap was used for reconstruction in 12 patients, and a fibular flap was used in 8 patients. In half of the patients, electrocautery and surgical clips were used for free flap harvesting (the EC group), and in the other half of the patients, ultrasonic dissection was performed using the Harmonic Scalpel (the HS group). The following parameters were significantly lower in the HS group compared to the EC group: the operative time of flap dissection (35% lower in the HS group), blood loss, number of surgical clips and cost of surgical materials. This study demonstrated the effectiveness of the Harmonic Scalpel in forearm and fibular free flap dissections that may be extended to other free flaps.

## 1. Introduction

Free tissue transfers are widely performed to reconstruct head and neck defects, often after the resection of head and neck cancer. Free tissue transfers allow the tissue volume to recover and improve functional and esthetic results. Radial forearm and composite fibular flaps are the two most frequent free flaps used in head and neck reconstructive surgery. To decrease the operative time, surgical procedures generally involve two teams: one team removes the tumor, and the other team performs the reconstruction.

Surgeons commonly use electrocautery dissection and surgical clip appliers for free flap tissue dissection. New ultrasonic dissection surgical techniques have been developed that use instruments to convert high-frequency ultrasonic waves (55,000 Hz) into mechanical energy. With ultrasonic dissection, surgical dissection and hemostasis of small to medium sized vessels are performed using the same surgical instrument by disrupting hydrogen bonds and forming coagulum. Ultrasonic dissection was initially used in gastrointestinal surgery [1–3] and urology and is now widely used in many surgical specialties, including plastic and reconstructive surgery (i.e., in abdominal lipectomies [4], face lifts [5], and myocutaneous flaps [6, 7]).

The aim of this study was to evaluate the effectiveness and advantages of the Harmonic Scalpel (Harmonic Synergy Curved Blade) in free flap surgery. We report on our experience with radial forearm and fibular flap harvesting in 20 patients and compare the electrocautery dissection performed in half of the patients to the ultrasonic dissection performed in the other half of patients. Several parameters for effectiveness and cost were analyzed and statistically compared between the two dissection procedures.

## 2. Patients and Methods

This prospective study recruited patients between March 2009 and May 2010. Head and neck tissue reconstruction was performed in 20 patients undergoing radial forearm or fibular free flap dissection. Most (14/20) of the patients were treated for head and neck carcinoma, three were treated for osteoradionecrosis, one was treated for mandibular ameloblastoma, one for mandibular osteitis, and one for postoperative pharyngeal fistula. Surgery was performed after prior radiotherapy treatment in 45% (9/20) of the patients.

To decrease the duration of the operation, the surgical procedure involved two teams: one team performed the cancer resection, and the other team raised the free flap and performed the reconstruction. The same surgeon performed all of the free flap dissections and reconstructions. In all of the patients, free flap tissue dissection was performed after exsanguination using an elastic bandage and a pneumatic tourniquet. Head and neck reconstruction required a forearm free flap in 12 patients and a fibular free flap in 8 patients. In half of the patients, electrocautery was used for free flap tissue dissection, and the vessels were controlled with electrocautery or surgical clips (Electrocautery and Clips: the EC group) (Surgical Clip Applier: Ligaclip MCM20, Ethicon, Endosurgery Inc., Cincinnati, OH). In the other half of the patients, ultrasonic dissection was performed using the Harmonic Scalpel (Harmonic Synergy Curved Blade, Ethicon Endosurgery Inc., Cincinnati, OH) for both tissue dissection and hemostasis (Harmonic Scalpel: the HS group). Vessel hemostasis was performed in the HS group using the anterior or posterior side of the Harmonic Curved Blade when the vessel diameter was less than 3 mm. A section was obtained with the same instrument using the cutting segment at the periphery of the Harmonic Curved Blade. If the diameter of the vessel was greater than 3 mm, medium surgical clip appliers were used. To compare the two surgical dissection techniques, several parameters were analyzed, including operative time for the flap dissection (related to the tourniquet time), volume level in the drains after dissection, number of surgical clip appliers, the mean cost per patient of disposable surgical material used for free flap harvesting (cost of surgical clip appliers used + cost of the Harmonic Synergy Curved Blade, in Euros in reference to the price in France), and postoperative complications.

Statistical analysis was performed using an unpaired, two-tailed Student's *t*-test. A value of *P* < 0.05 was considered to indicate a significant difference between two groups.

## 3. Results

Twenty patients were included in the study. The male : female ratios in the HS and EC groups were 7 : 3 and 8 : 2, respectively. The mean age of the patients was 57.2 years (range: 27 to 71 years) in the HS group and 57.9 years (range: 48 to 69 years) in the EC group. 

**Figure 1:**
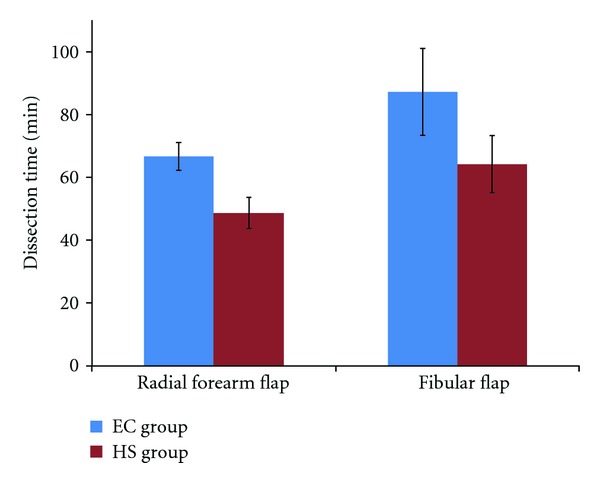
Flap harvesting time (in min) in each group (HS and EC groups).

**Figure 2:**
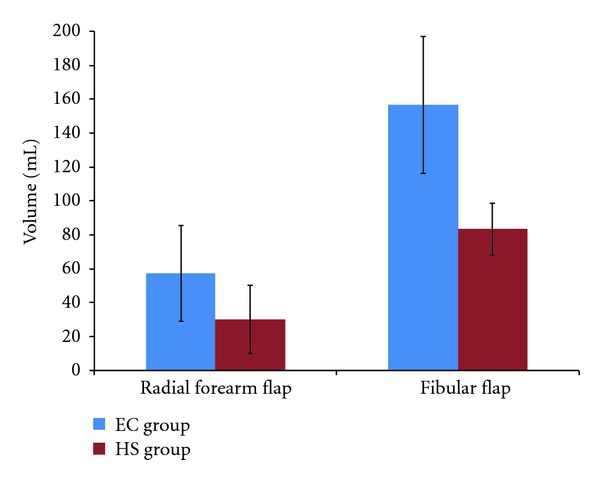
Postoperative cumulative volume in drains (in mL) in each group (HS and EC groups).

The statistical results of the analyzed parameters used to compare the HS and EC groups are showed in Table 1 and Figures 1 and 2. The mean operative time of flap dissection (related to the tourniquet time) was significantly lower (35%) in the HS group compared to the EC group: respectively, 55 min and 75 min. The HS group demonstrated significantly less blood loss. The postoperative blood loss (cumulative volume of drains) was less significant overall. The number of surgical clip appliers was lower in the HS group; therefore, considering the specific material used for the free flap harvesting, the mean operative cost was significantly lower in this group (511 Euros versus 1,021 Euros). 

All of the free flaps survived, and the morbidity of the reconstruction and donor sites was not statistically significant between the groups (wound infection, hematoma, skin graft diseases, and tissue retraction: *P* > 0.05).

## 4. Discussion

The use of free flaps is considered to be a standard procedure in head and neck reconstruction after cancer resection involving composite tissues. Free flaps provide superior cosmetic and functional restoration compared to other flaps, with limited donor site morbidity. The most common free flaps in head and neck reconstruction are forearm and fibular free flaps. Radial forearm flaps are used in the reconstruction of mucous, membranes and muscles of the oral cavity, oropharynx and hypopharynx, as well as in large skin defects of the face. Fibular free flaps are used for the reconstruction of bone and adjacent tissues (i.e., the mandible and maxilla). The length of the surgery is increased by the requirement for many stages: removal of the tumor, harvesting of the free flap, preparation of the recipient vessels, microsurgical anastomoses, and reconstruction. Therefore, to decrease operative time, surgical procedures generally involve two teams: one team removes the tumor, and the other team performs the reconstruction. The utilization of ultrasonic dissection is practical for the surgeon because of the quality and speed of this procedure of tissue dissection, which also minimizes thermal damage to tissues.

Because it is important to preserve the quality of the flap and the donor site tissues, ultrasonic dissection may influence the quality of reconstruction and healing. Many studies have reported a decrease in seroma formation with the Harmonic Blade in plastic and reconstructive surgery [4, 6–9]. The temperature generated by the Harmonic Scalpel is much lower than that generated in conventional electrocautery; therefore, lateral tissue destruction is much lower using ultrasonic dissection [10, 11]. In this study, the mean operative time was significantly shorter (35%) in patients treated with the Harmonic Scalpel compared to electrocautery: respectively, 55 min and 75 min. This decreased time was important in reducing the length of pneumatic tourniquet application, which is often used to facilitate forearm and fibular flap dissection. Therefore, it may reduce the risk of nerve-related injury.

Interestingly, the Harmonic Scalpel allows a single surgical instrument to be used for tissue dissection, hemostasis, and sectioning of vessels. In this study, postoperative fluid collection in drains (due to bleeding and seroma formation) was significantly higher in the fibular flap subgroup using HS and statistically equivalent in the forearm flap subgroup. Nevertheless, concerning the forearm subgroup, the volume of drains was lower in the HS group, with a *P* value of 0.056, which implies that these results could be statistically significant in a larger cohort of patients. Therefore, hemostasis quality was comparable or better with the Harmonic Scalpel. These results confirm the data from previous studies that clearly demonstrated better hemostasis using the Harmonic Scalpel, especially the results of a meta-analysis of thyroid surgery [12].

In this study, there was no significant difference between the Harmonic Scalpel and electrocautery in regard to the level of postoperative pain concerning the flap harvesting site. This parameter was difficult to analyze objectively because the two surgical sites used for each patient may have skewed the interpretation of pain. Previous studies have not found any difference between the type of scalpel used and the severity of postoperative pain [13–15]. Nevertheless, other studies have found significantly reduced postoperative pain using Harmonic Scalpel dissection in hemorrhoidectomy surgery compared to bipolar electrocautery [16], in tonsillectomy compared to standard dissection and electrocautery [17], or in neck lymphadenectomy [18]. These results could be explained by the avoidance of excessive lateral thermal injury caused by electrocautery. In this study, morbidity of the reconstruction site and donor site (wound infection, hematoma, skin graft diseases, and tissue retraction) was rare and statistically similar between the two groups.

The pedicle of the free flap requires many muscular perforators that must be ligated and divided using conventional surgical clips or bipolar cautery. Authors reported the use of bipolar cautery alone for perforator hemostasis, but, in our experience surgical clips appear more effective and secure. The Harmonic Scalpel offers the ability to perform the hemostasis of muscular perforators by using the anterior or posterior side of the Harmonic Synergy Curved Blade and by cutting the vessel with the lateral side of the blade. In this study, the mean numbers of surgical clip appliers were 7.4 in the EC group and 1.1 in the HS group. Therefore, the mean cost per patient of disposable surgical material (clip applier and/or Harmonic Synergy Curved Blade) in the EC and HS groups was 1,021 and 510 Euros, respectively. The cost in the EC group could be lower than that in the HS group if bipolar cautery was used instead of surgical clips, as described by other surgical teams. Nevertheless, our results are consistent with previous studies that reported the cost-effectiveness of the Harmonic Scalpel compared to conventional surgical dissection [3, 19, 20].

## 5. Conclusion

This study demonstrated the effectiveness of using the Harmonic Scalpel in forearm and fibular free flap harvesting compared to conventional surgical instruments. The Harmonic Scalpel provided a high quality of dissection and hemostasis, minimized tissue injuries, resulted in a 35% reduction in operative time, and reduced the mean operative cost. In this study, we reported the use of the Harmonic Scalpel in the dissection of forearm and scapula free flaps that could be extended to other free flaps.

## Figures and Tables

**Table 1:** Statistical results of the parameters analyzed to compare the HS and EC groups.

		HS group^1^	EC group^2^	
		Mean ± SD	Mean ± SD	*P *value
Flap dissection time (min)	Both freeflaps (*n* = 20)	55 ± 9.7	74.9 ± 13	<0.001
	Radial forearm flap (*n* = 12)	48.8 ± 4.6	66.7 ± 4.1	<0.001
	Fibular flap (*n* = 8)	64.3 ± 7.8	87.3 ± 11.9	0.018

Postoperative cumulative volume of drains (mL)	Both freeflaps	52.9 ± 30.6	90.4 ± 54.8	NS (0.063)
	Radial forearm flap	30 ± 17.3	57.3 ± 25.9	NS (0.056)
	Fibular flap	83.3 ± 12.5	156.7 ± 33	0.037

Cost of surgical materials*	Both freeflaps	510.8 ± 41.4	1021 ± 140.4	<0.001
	Radial forearm flap	497	1080 ± 123.9	<0.001
	Fibular flap	531.5 ± 59.8	931.5 ± 114.4	0.002

^1^HS group: flap dissection group using the Harmonic Scalpel; ^2^EC group: flap dissection group using electrocautery and surgical clips.

*Addition of the cost of the surgical clip appliers plus the Harmonic synergy Curved Blade in Euros (in reference to the price in France), according to the following method: (nb of clip appliers × 138 euros (=unitary price)) + 368 euros (Harmonic synergy curved blade price).

Postop: postoperative; vol.: volume; NS: nonsignificant (*P* > 0.05).
